# Serum Magnesium and Sudden Death in European Hemodialysis Patients

**DOI:** 10.1371/journal.pone.0143104

**Published:** 2015-11-23

**Authors:** Camiel L. M. de Roij van Zuijdewijn, Muriel P. C. Grooteman, Michiel L. Bots, Peter J. Blankestijn, Sonja Steppan, Janine Büchel, Rolf H. H. Groenwold, Vincent Brandenburg, Marinus A. van den Dorpel, Piet M. ter Wee, Menso J. Nubé, Marc G. Vervloet

**Affiliations:** 1 Department of Nephrology, VU University Medical Center, Amsterdam, the Netherlands; 2 Institute for Cardiovascular Research, VU University Medical Center (ICaR-VU), VU University Medical Center, Amsterdam, the Netherlands; 3 Julius Center for Health Sciences and Primary Care, University Medical Center Utrecht, Utrecht, the Netherlands; 4 Department of Nephrology, University Medical Center Utrecht, Utrecht, the Netherlands; 5 Fresenius Medical Care Deutschland GmbH, Bad Homburg, Germany; 6 Department of Cardiology and Vascular Medicine, University Hospital Aachen, Aachen, Germany; 7 Department of Internal Medicine, Maasstad Hospital, Rotterdam, the Netherlands; University of São Paulo School of Medicine, BRAZIL

## Abstract

Despite suggestions that higher serum magnesium (Mg) levels are associated with improved outcome, the association with mortality in European hemodialysis (HD) patients has only scarcely been investigated. Furthermore, data on the association between serum Mg and sudden death in this patient group is limited. Therefore, we evaluated Mg in a *post-hoc* analysis using pooled data from the CONvective TRAnsport STudy (CONTRAST, NCT00205556), a randomized controlled trial (RCT) evaluating the survival risk in dialysis patients on hemodiafiltration (HDF) compared to HD with a mean follow-up of 3.1 years. Serum Mg was measured at baseline and 6, 12, 24 and 36 months thereafter. Cox proportional hazards models, adjusted for confounders using inverse probability weighting, were used to estimate hazard ratios (HRs) of baseline serum Mg on all-cause mortality, cardiovascular mortality, non-cardiovascular mortality and sudden death. A generalized linear mixed model was used to investigate Mg levels over time. Out of 714 randomized patients, a representative subset of 365 (51%) were analyzed in the present study. For every increase in baseline serum Mg of 0.1 mmol/L, the HR for all-cause mortality was 0.85 (95% CI 0.77–94), the HR for cardiovascular mortality 0.73 (95% CI 0.62–0.85) and for sudden death 0.76 (95% CI 0.62–0.93). These findings did not alter after extensive correction for potential confounders, including treatment modality. Importantly, no interaction was found between serum phosphate and serum Mg. Baseline serum Mg was not related to non-cardiovascular mortality. Mg decreased slightly but statistically significant over time (Δ -0.011 mmol/L/year, 95% CI -0.017 to -0.009, *p* = 0.03). In short, serum Mg has a strong, independent association with all-cause mortality, cardiovascular mortality and sudden death in European HD patients. Serum Mg levels decrease slightly over time.

## Introduction

Despite continuous research as well as improvements in treatment, mortality among hemodialysis (HD) patients remains unacceptably high [1], of which a large proportion can be attributed to cardiovascular causes [2]. Magnesium (Mg) is the fourth most abundant cation in the human body. Its role, however, has been neglected in HD patients. Mg is involved in a wide variety of biological processes [3], such as inhibition of vascular calcification [4–6] and cation fluxes in cardiomyocytes [7].

In non-renal cohorts, low serum Mg is associated with atherosclerotic lesions [8], coronary disease [9], and sudden death [10]. Furthermore, data suggests that a higher Mg intake is associated with decreases of aortic and carotid arterial calcification scores [11,12].

In Japanese HD patients, serum Mg was recently shown to be inversely associated with all-cause, cardiovascular and non-cardiovascular mortality [13,14]. In addition, these researchers demonstrated that Mg levels modify the risk of serum phosphate on adverse clinical outcomes in the same large cohort, i.e. a significant decrease in cardiovascular mortality risk in patients with a high phosphate when serum Mg levels increase [15]. Most of these associations were identified in the Japanese population, which is fundamentally different from western people with regard to medical history, such as dialysis vintage or transplantation rate [16,17], and dietary habits, including Mg intake [18]. Nevertheless, two recent American studies and a Portuguese study confirmed the inverse association between serum Mg and all-cause mortality in HD patients. However, generalizability from both Japanese and American studies to European HD patients warrants confirmation of the findings. Furthermore, the Portuguese study is limited by a binary approach of the serum Mg level and a hypermagnesemic population (mean serum Mg 1.36 mmol/L versus a reference range of 0.70–1.00 mmol/L in the Netherlands) [19–21].

Thus, the question arises whether the positive association between serum Mg and survival can be confirmed in a large, well-defined European HD cohort. Furthermore, presently, limited data are available on the association between serum Mg and sudden death in HD patients as well as data concerning serum Mg levels over time. To elucidate these issues, we analysed samples of patients from the CONvective TRAnsport STudy (CONTRAST).

## Materials and Methods

CONTRAST was conducted in accordance with the declaration of Helsinki and Good Clinical Practice guidelines and was approved by the central medical ethics review board of VU University Medical Center, Amsterdam, the Netherlands (METC VUmc 2003/97). The design and methods of the CONTRAST study (NCT00205556) have been described elsewhere [22,23]. In brief, CONTRAST was an RCT designed to evaluate the effect of postdilution online hemodiafiltration (HDF) compared to low-flux HD on all-cause mortality and cardiovascular events. From 2004–2010, 714 patients were enrolled in 29 dialysis facilities in 3 countries (the Netherlands [n = 26], Canada [n = 2] and Norway n = 1]). Adult (≥18 years) end-stage kidney disease (ESKD) patients were eligible when treated chronically (≥2 months) with HD two or three times per week. Furthermore, patients had to be able to understand the study procedures and be willing to provide informed consent. Exclusion criteria were severe incompliance to dialysis prescription, treatment with HDF or high-flux HD in the 6 months preceding randomization, participation in another clinical intervention trial evaluating cardiovascular outcome or a life expectancy below 3 months due to a non-renal disease. Written informed consent was obtained from all participants prior to randomization.

### Patients

In a subset of centers in which it was logistically feasible to collect and store blood samples for non-routine assessment (only Dutch and Norwegian patients), serum samples were drawn prior to dialysis and stored at -80 degrees Celsius for future determinations. These samples were collected at baseline and 6, 12, 24 and 36 months thereafter. Mg (total serum Mg^++^) was measured in 2014 in these samples in a central laboratory (VU University Medical Center, Amsterdam, the Netherlands) photometrically through the decrease in xylidyl blue absorption. Participants from CONTRAST were included in the present analysis if at least baseline serum Mg had been measured. Data from the two treatment arms (low-flux HD and postdilution online HDF) were pooled for this study.

### Data collection

Demographic data, medical history, dialysis characteristics, routine laboratory measurements and medication use were recorded at baseline. Subsequently, routine laboratory measurements, medication use and dialysis characteristics were registered every three months thereafter. In both study arms, patients were treated with bicarbonate-containing ultrapure dialysis fluid, of which the Mg concentration is usually 0.5 mmol/L.

### Follow-up

Patients were followed from randomization until death or end of the study. Clinical events were continuously monitored. There was no loss to follow-up as patients who stopped the randomized treatment, for example due to renal transplantation, a switch to peritoneal dialysis or moving to a non-participating center, were still followed for mortality. Cardiovascular death was defined as death due to myocardial infarction, rupture of an aneurysm, sudden death, terminal heart failure or either hemorrhagic or ischemic stroke. Sudden death was defined as either certain sudden death (death within one hour after onset of symptoms as verified by a witness) or probable sudden death (death within 24 hours after onset of symptoms as verified by a witness or found dead by a witness). An independent Endpoint Adjudication Committee reviewed source documentation of all clinical outcomes.

### Statistical analyses

As the number of one of the outcome parameters was limited (sudden death; n = 24), an inverse probability weighted marginal structural model (IPWMSM) with Mg as continuous exposure was used to be able to correct for more confounders than allowed by conventional adjustment for confounding by regression analysis [24]. Using the R-package ‘ipw’, for each individual weights were estimated based on information of various confounders (https://cran.r-project.org/web/packages/ipw/index.html). Weighting each participants by his or her weight then results in a weighted population in which Mg levels are independent of confounding values. Next, weighted Cox proportional hazards models were fitted with a robust variance estimator to estimate hazard ratios (HRs) per 0.1 mmol/L increase in serum Mg. Five models were fitted: a crude model and four models adjusted for a priori selected potential confounders. In the first adjusted model, HRs were corrected for age, sex, dialysis vintage, and residual kidney function. The second model was corrected for the confounders in model 1 plus diabetes, BMI, history of cardiovascular disease, and dialysis modality (HD/HDF). In the third model, HRs adjusted for the potential confounders of model 2 plus serum albumin, the mean pre-dialytic systolic blood pressure, and treatment time were estimated. The last model calculated HRs corrected for the confounders in model 3 plus serum calcium, serum parathyroid hormone and serum phosphate. To check whether the results of the analysis adjusting with an IPWMSM are valid, as a comparison, Cox proportional hazards models were also fitted with all confounders included as covariates into the model. For all-cause mortality, a sufficient number of events occurred (n = 137) to be able to appropriately fit a multivariable Cox regression model, allowing this model to be compared with the IPWMSM model. The assumptions of the Cox proportional hazards models, i.e. proportional hazards and a linear association between Mg and mortality hazard, were checked with Martingale and Schoenfeld residuals, respectively, and appeared to hold.

Possible interaction between serum Mg and serum phosphate in relation to clinical outcome was also explored [15]. Cox regression analysis was used to fit twelve models (4 categories as described below and 3 end points: all-cause mortality, cardiovascular mortality and sudden death). First, three models were fitted with serum Mg as a continuous variable, serum phosphate as a dichotomous variable and an interaction term between these variables. Hereafter, similar models were fitted using serum Mg as a dichotomous variable, serum phosphate as a continuous variable and an interaction term. Third and fourth, models with both variables as continuous values with an interaction term and both variables as dichotomous variables with an interaction term were fitted, respectively.

For the longitudinal data on Mg evolution, a generalized linear mixed model was used with either a random intercept or a random slope plus a random intercept, based on the lowest Aikaike’s Information Criterion. As the time between measurements was unequal (i.e. either 6 or 12 months), a continuous autoregressive covariance matrix was used. Lastly, the entire cohort was divided into quartiles based on their baseline Mg concentration. The course over time from these 4 groups was plotted to visualize any possible regression to the mean. Statistical analyses were performed using either the statistical software package SPSS 20.0 (IBM Inc., IL, USA) or RStudio (RStudio Inc., version 0.98.932).

### Sensitivity analysis

As a sensitivity analysis, the aforementioned analyses were repeated with censoring of patients at the time of treatment discontinuation due to, for example, a switch to peritoneal dialysis, moving to a non-participating center or renal transplantation. Again, crude and adjusted Cox proportional hazards models were used to calculate HRs per 0.1 mmol/L increase in serum Mg.

## Results

### Patients and magnesium distribution

**Fig 1:**
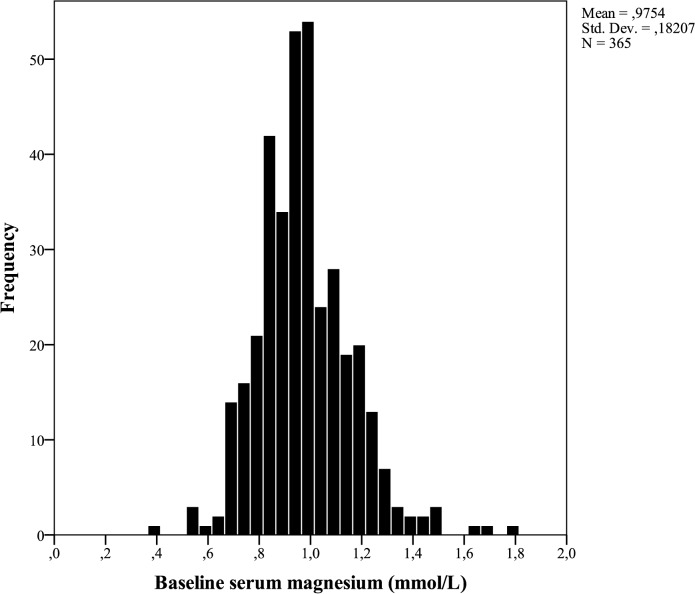
Distribution of baseline serum magnesium (mmol/L).

Out of the 714 patients included in CONTRAST, baseline serum Mg was available for 365 Dutch and Norwegian patients. Baseline characteristics of both the entire cohort and the investigated cohort are shown in Table 1. No marked differences were observed between these groups. In the investigated patients, mean age was 63.5±13.8 years and 61.9% were male. Mean serum Mg was 0.98±0.13 mmol/L (Fig 1). Of note, the baseline Mg value of patients randomized to HD was slightly lower than in patients randomized to HDF (mean 0.95±0.17 and 1.00±0.19 mmol/L, respectively, *p* for difference = 0.01). Mean spKt/V_urea_ was 1.39±0.20 per dialysis session and mean follow-up was 3.06±1.76 years.

**Table 1:** Baseline Patient Characteristics.

Determinant	Investigated cohort (n = 365)	Entire cohort (n = 714)
Age (years)	63.5 (13.8)	64.1 (13.7)
Sex (male)	226 (61.9%)	445 (62.3%)
BMI (kg/m^2^)	24.9 (4.9)	24.7 (4.8)
Region (NL)	350 (95.9%)	597 (83.6%)
Diabetes	76 (20.8%)	170 (23.8%)
Cardiovascular disease	163 (44.7%)	313 (43.8%)
Residual kidney function*	212 (58.1%)	376 (52.7%)
Previous renal transplantation	41 (11.2%)	78 (10.9%)
Phosphate (mmol/L)	1.67 (0.52)	1.64 (0.49)
Albumin (g/L)^#^	39.9 (4.0)	40.4 (3.8)
Cholesterol (mmol/L)	3.66 (0.99)	3.68 (0.96)
Creatinine (μmol/L)	864 (253)	861 (255)
Hemoglobin (g/L)	119 (13)	118 (13)
Magnesium (mmol/L)	0.98 (0.18)	NA
RAS inhibitor^¶^	201 (55.1%)	351 (49.2%)
Beta-blocker	210 (57.5%)	381 (53.4%)
Calcium antagonist	125 (34.2%)	230 (32.2%)
Statin	186 (51.0%)	369 (51.7%)
Platelet aggregation inhibitor	103 (28.2%)	240 (33.6%)
Dialysis vintage (years)	1.8 (0.9–3.4)	2.0 (1.0–4.0)
spKt/V_urea_	1.39 (0.20)	1.40 (0.22)
Assigned to HDF treatment	181 (49.6%)	358 (50.1%)

Data are presented as mean (standard deviation), median (interquartile range) or number (percentage), when appropriate

* Defined as diuresis >100 mL/24h

^#^ Bromocresol green values

^¶^ Prescription of either an ACE inhibitor or an ATII antagonist

Abbreviations: BMI = Body Mass Index; NL = the Netherlands; PTH = parathyroid hormone; CRP = C-Reactive Protein; RAS = renin-angiontensin system; HDF = hemodiafiltration; NA = not applicable

### Magnesium and mortality outcomes

**Fig 2:**
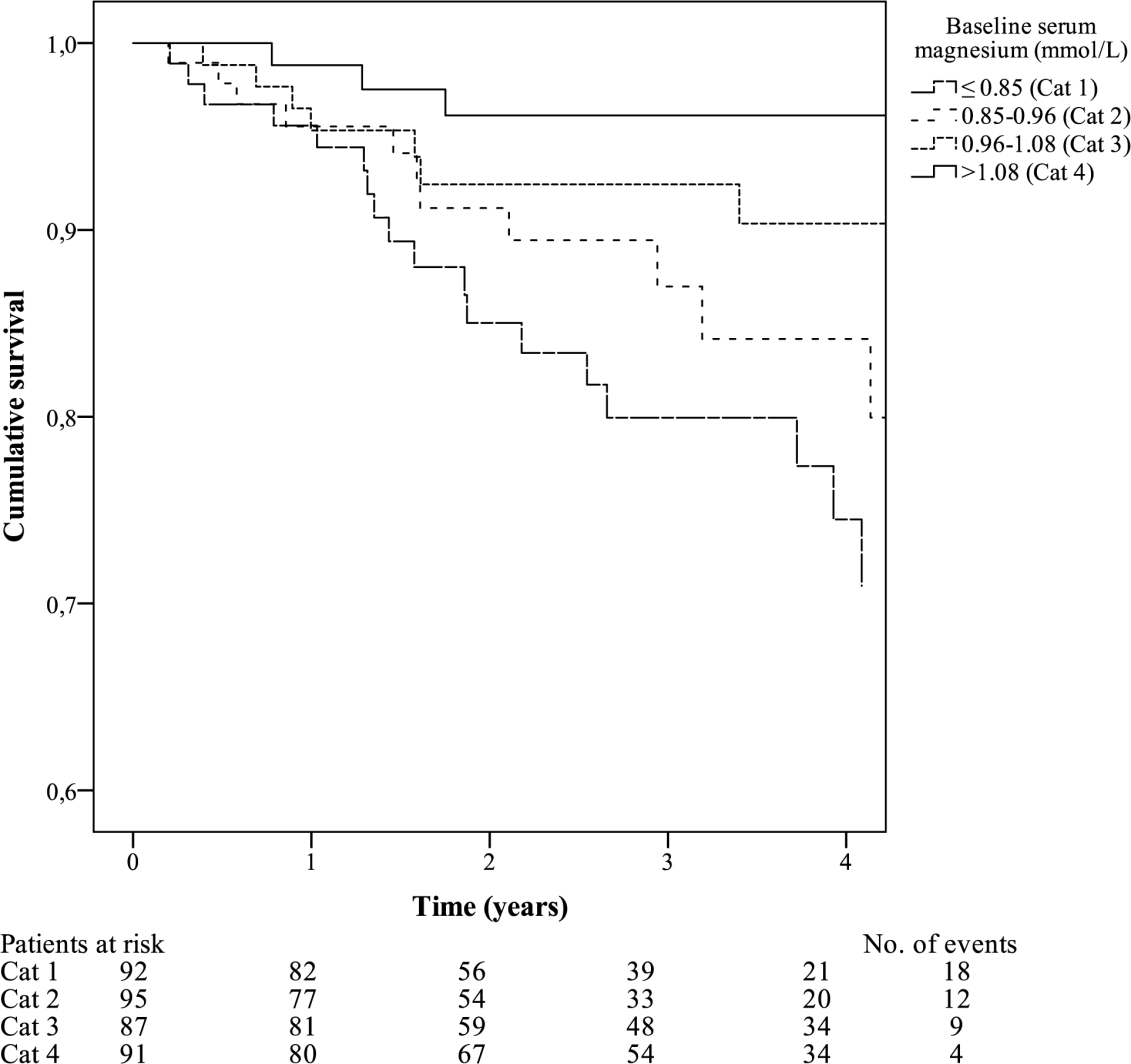
Cardiovascular survival curves stratified by quartiles of baseline serum magnesium. Kaplan-Meier curves of cardiovascular survival as stratified by baseline serum magnesium: ≤0.85 (cat 1, n = 92); 0.85–0.96 (cat 2, n = 95); 0.96–1.08 (cat 3, n = 87); >1.08 (cat 4, n = 91).

A linear association was found between serum Mg and all-cause mortality risk with a HR of 0.85 (95% CI 0.77–0.94), indicating a significant reduction in all-cause mortality risk of 15% per 0.1 mmol/L increase in serum Mg. For cardiovascular mortality, the association was even stronger: crude HR 0.73 (95% CI 0.62–0.85) per 0.1 mmol/L higher serum Mg concentration. The survival risk for cardiovascular mortality per Mg quartile was visualized with a Kaplan-Meier curve (Fig 2). A similar reduction in hazard for sudden death was found (crude HR 0.76 [95% CI 0.62–0.93]). Importantly, subsequent models adjusting for various upfront determined potential confounders did not materially change the strength of any of the associations. Details of the weighted Cox proportional hazards models are shown in Table 2. Results from the multivariable Cox regression model as a sensitivity analysis for the IPWMSM are shown in S1 Table. As can be seen from the comparison between Table 2 and S1 Table, the results from the different models were similar, suggesting that the IPWMSM is at least equally as valid as the conventional method to control for confounding. The results of the sensitivity analysis are shown in S2 Table, which show similar results. Importantly, none of the models exploring interaction between serum Mg and phosphate showed a significant result (*p*-values ≥0.32).

**Table 2:** Association between serum Mg and outcomes: results of weighted Cox proportional hazards models^#^.

	All-cause mortality (137 events)	Cardiovascular mortality (43 events)	Non-cardiovascular mortality (94 events)	Sudden death (24 events)
Crude*	0.85 (0.77–0.94)^&^	0.73 (0.62–0.85)^&^	0.91 (0.81–1.01)	0.76 (0.62–0.93)^&^
Model 1^^^	0.87 (0.79–0.96)^&^	0.74 (0.64–0.86)^&^	0.93 (0.83–1.04)	0.77 (0.64–0.94)^&^
Model 2^$^	0.85 (0.77–0.94)^&^	0.73 (0.62–0.86)^&^	0.91 (0.81–1.01)	0.77 (0.62–0.94)^&^
Model 3^¶^	0.88 (0.79–0.98)^&^	0.74 (0.64–0.86)^&^	0.93 (0.83–1.05)	0.79 (0.66–0.94)^&^
Model 4^§^	0.88 (0.78–0.99)^&^	0.73 (0.62–0.85)^&^	0.93 (0.82–1.06)	0.78 (0.66–0.92)^&^

Results presented as hazard ratios (HRs [95% confidence intervals]) per 0.1 mmol/L higher baseline serum magnesium concentration. Adjusted for confounders described below using an ^#^inverse probability weighted marginal structural model

* Crude model (n = 365)

^^^ Adjusted for age, sex, dialysis vintage and residual kidney function (n = 365)

^$^ Adjusted as in model 1 plus diabetes mellitus, BMI, history of cardiovascular disease and dialysis modality (n = 351)

^¶^Adjusted as in model 2 plus serum albumin, mean pre-dialytic systolic blood pressure and treatment time (n = 339)

^§^ Adjusted as in model 3 plus serum calcium, serum parathyroid hormone and serum phosphate (n = 334)

^&^ indicates a significant difference in adverse event risk (*p*<0.05)

### Magnesium evolution over time

**Fig 3:**
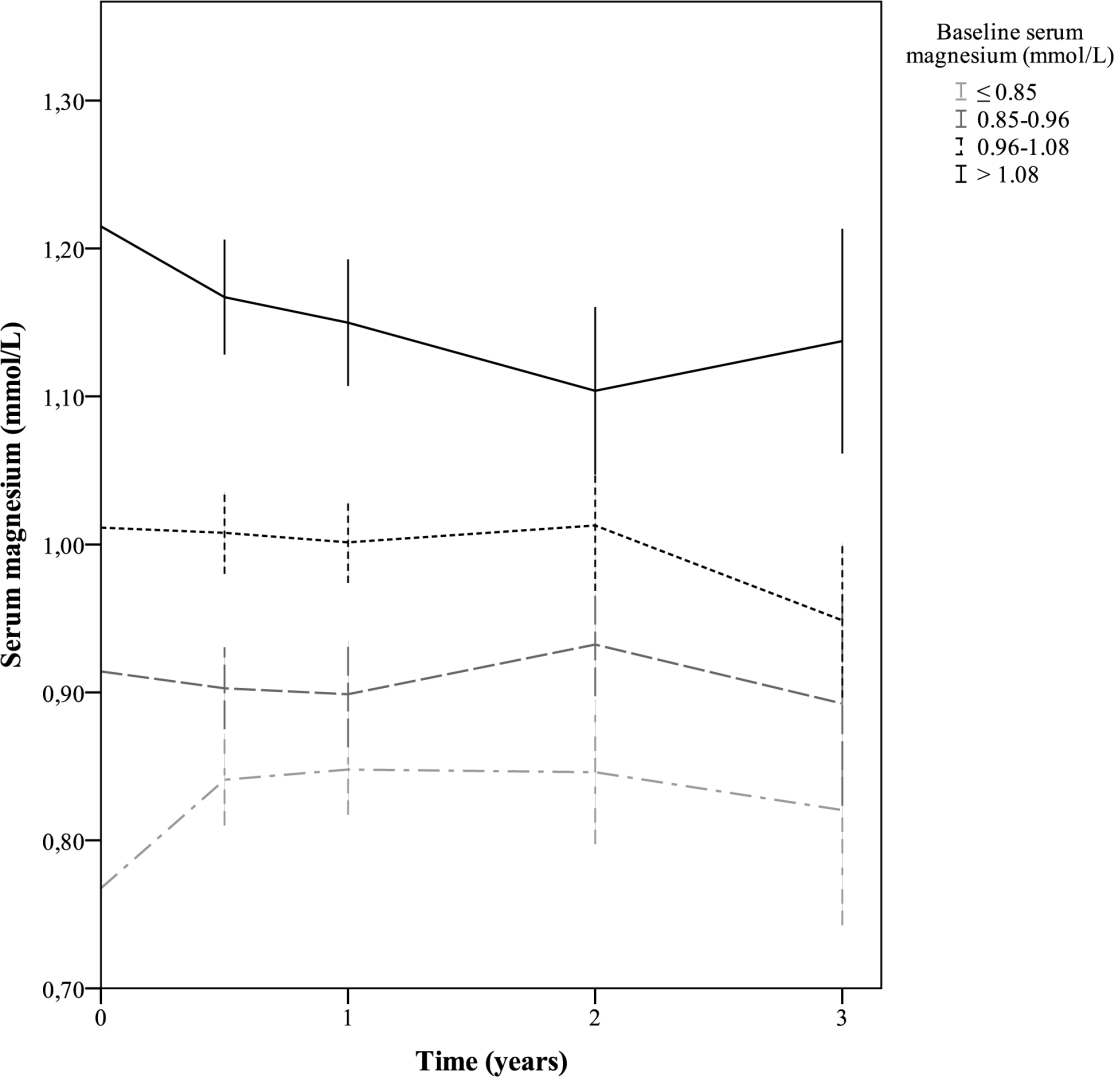
Serum magnesium over time stratified by baseline serum magnesium quartiles. Serum magnesium concentration over time with 95% confidence intervals at baseline and 6, 12, 24 and 36 months thereafter, as stratified by quartiles of baseline serum magnesium: ≤0.85 (cat 1, n = 92); 0.85–0.96 (cat 2, n = 95); 0.96–1.08 (cat 3, n = 87); >1.08 (cat 4, n = 91).

From the generalized linear mixed model, it was shown that on average serum Mg decreased over time in these patients (Δ -0.011 mmol/L/year, 95% CI -0.017 to -0.009, *p* = 0.03). Longitudinal data as stratified by baseline serum Mg quartiles were plotted (Fig 3). As can be seen from this graph, the serum Mg concentration quartile slopes do not cross during follow-up.

## Discussion

In the present study, a strong and inverse association was found between baseline serum Mg and all-cause mortality, cardiovascular mortality and sudden death. The risk on these adverse events drops linearly per 0.1 mmol/L higher serum Mg. Importantly, extensive correcting for potential confounders did not at all mitigate the strength of any association. The large number of measured confounders that was adjusted for suggest that the impact of confounding on the association was probably small. Furthermore, we showed that serum Mg in HD patients decreases slightly over time. As serum Mg in dialysis patients depends on intake, uptake, residual kidney function and the Mg concentration of the dialysis fluid, this important risk factor is potentially modifiable. As such, the current study sets the stage for prospective intervention trials to test the hypothesis that the high mortality rate in HD patients can be improved by increasing their serum Mg concentration.

An observational study on 515 Japanese HD patients found that in an adjusted time-to-event analysis, the HR was 0.49 (95% CI 0.24–0.98) per 1.0 mg/dL (0.41 mmol/L) higher serum Mg [13]. In that study, the effect was most pronounced for non-cardiovascular mortality, which is opposite to previous findings of studies in non-renal patients and to the current study. A more recent logistic regression analysis from Japan on almost 150000 HD patients showed that the fifth sextile of Mg (2.8–3.1 mg/dL [1.15–1.27 mmol/L]) had the lowest odds on both cardiovascular and non-cardiovascular mortality [14]. A large American study found an almost linear association between serum Mg and death [19], and another American cohort study confirmed the inverse relation between serum Mg and survival [20]. Lastly, a Portuguese study found similar results in a hypermagnesemic cohort where serum Mg was dichotomized [21]. Some of these findings differ from our study, which may be explained by adjustment for other covariates, different population characteristics, or both. Oriental and occidental dietary habits, including Mg intake, differ [18] and probably influence outcome. Furthermore, the transplantation rate in Japan is much lower compared to Europe [17]. As in Europe the younger and healthier patients tend to get transplanted, an older population remains on dialysis in this geographic area. Consequently, the dialysis population in Japan is generally younger and in a better shape when compared to the European dialysis population. These hypotheses are supported by the fact that in the first Japanese study, 103/515 patients died (20%) over a mean follow-up of 51 months, while in our study 137/365 patients died (37.5%) over a mean follow-up of 37 months. Furthermore, in the large database-driven study of HD patients from Japan, mean serum Mg was 2.61±0.52 mg/dL (1.07±0.21 mmol/L), whereas the mean serum Mg concentration was 0.98±0.13 mmol/L at baseline in our study. Finally, the Mg concentration in the highest sextile from the study in Japan (above 3.1 mg/dL [1.27 mmol/L]) was rare in our population. Therefore, we cannot investigate whether the hazard for survival is lower at the higher extreme end of Mg concentrations in western HD populations as well. Our findings concerning survival are in line with the recent studies on 27544 and 9359 prevalent and incident American HD patients [19,20] as well as the findings from João Matias *et al* [21].

The strong association between Mg and cardiovascular mortality raises the question of the possible biological mechanisms involved. In patients with chronic kidney disease (CKD), the transformation of vascular smooth muscle cells (VSMCs) to an osteochrondrogenic phenotype is involved in arterial medial calcification (AMC) [25], increasing arterial stiffness. Patients with AMC have an impaired prognosis compared to patients without arterial calcification [26,27]. In ESKD, it has been suggested that a mild hypermagnesemia may retard the development of arterial calcification [4]. In bovine VSMC, calcification induced by ß-glycerophosphate could be prevented by co-incubation with Mg in a dose-dependent matter. Furthermore, with higher Mg levels, an inhibited expression of osteogenic proteins, apoptosis and further progression of already established calcification was found [5]. Moreover, in the presence of Mg, less calcification of aortic segments of rats was observed despite increasing phosphate levels [6], an effect that was confirmed in human aorta VSMCs [28] and in line with the recent finding of a reduced cardiovascular mortality risk with increasing Mg in patients with a high phosphate level [15]. Possibly, reductions in the phosphate-induced Wnt/ß-catenin pathway activation partly drives these findings [29]. In this respect, however, it should be noted that in contrast to the Japanese findings in our cohort no interaction between Mg and phosphate was found, which would necessitate further research on the possible role of phosphate in the association between serum Mg and clinical outcome.

Recent evidence showed a positive association between Mg and flow-mediated dilatation (FMD), a measurement of endothelial functioning, in CKD patients, which is consistent with the negative association between Mg and vascular stiffening [30]. Lastly, a double-blind placebo-controlled RCT of non-renal patients with coronary artery disease (CAD) showed that supplementation of Mg improved FMD [31]. Altogether, Mg may play a protective role in the process of vascular calcification, which could be an explanation of the inverse association between cardiovascular mortality and serum Mg in this analysis.

Interestingly, we also found an association between serum Mg and sudden death, the single most important cause of death in western dialysis populations. These findings are in line with a previous study investigating sudden death in a HD cohort with a follow-up of 1 year [19]. In a double-blind, placebo-controlled RCT of Mg supplementation in CAD patients, an improvement in exercise tolerance and a decrease in ischemic ST-segment changes was observed in the intervention group [31]. *In vitro*, vasodilatation decreased in coronary segments in the absence of Mg and improved again when Mg was replenished. Blockade of nitric oxide (NO) release was suggested as the causal mechanism [32]. Lastly, Mg plays an essential role in the de- and repolarisation of cardiac smooth muscle cells [7]. Hence, it is appealing to speculate that Mg critically influences heart rhythm stability.

As can be seen from our results, serum Mg decreased statistically significant with 0.011 mmol/L/year. However, whether this decrease is clinically relevant is at least debatable given the fact that this decrease per year is only 10% of the difference in serum Mg concentration that was associated with survival in this cohort. Importantly, as can be seen from Fig 3, patients in the upper range of serum Mg remain in the upper region. Hence, the rate of change found was not the result of regression to the mean.

As mentioned, Mg supplementation may improve clinical outcome. In many countries, a Mg dialysis fluid concentration of 0.5 mmol/L (1.0 mEq/L) is standard and this level could easily be increased. Furthermore, dietary advices [12] or prescription of Mg-containing phosphate binders [33,34] are other potential options to increase a patients’ Mg level. Obviously, the Mg concentration has to be carefully monitored in such interventions because our data cannot prove safety of very high Mg levels.

The main strengths of the present study are the relative long follow-up, the precise and prospective collection of data, including Mg and follow-up of subjects that left the original study, and the review of end point source documentation for all primary outcomes. Fourth, the serial measurements of Mg in a central laboratory increases the reliability of the Mg measurement. Furthermore, the validity of the Cox regression models adjusted with an IPWMSM was checked in the model for all-cause mortality. From this multivariable analysis, the validity of the statistical model used is underscored. Lastly, the sensitivity analysis with censoring at the time of treatment discontinuation increases the robustness of our findings. A limitation is the availability of baseline serum Mg in only half the patients from the CONTRAST cohort due to logistical constraints. Even though the baseline characteristics appear similar between the entire cohort and the investigated cohort, a selection bias cannot be fully excluded. Second, given the observational nature of the present study, no causal relations can be drawn from this analysis as correction for unknown or unmeasured confounding is impossible. Given the association between serum Mg and sudden death, the absence of serum potassium in the adjusted models necessitates exploration of this particular finding in an external cohort. Furthermore, confounding by healthy dietary habits should be considered. However, it may be possible that beneficial effects of dietary habits are in fact driven by Mg itself. In this respect, it should also be noted that no correction was able to attenuate the considerable association between Mg and mortality. Lastly, the association between baseline serum Mg and sudden death seems peculiar since the time between measurement and event may be several years. However, as serum Mg did not materially change over time as was shown by linear mixed models and visualized in Fig 3, our findings seem reliable. Nonetheless, a future prospective investigation should study Mg levels closer to an event to confirm our findings.

In short, we found a strong, independent association between serum Mg and all-cause mortality, cardiovascular mortality and sudden death in a western HD cohort. Furthermore, serum Mg in HD patients decreases slightly over time, but in view of the very small quantity of the decrease, the clinical relevance of this rate of change is uncertain. Future research should investigate determinants of serum Mg and determinants of its course over time. Furthermore, the promising potential causal mechanisms underlying this association are interesting topics for future research. Mg supplementation, either orally or by modifying Mg content of the dialysis fluid, appears to be an easy tool to perform such trials.

## Supporting Information

S1 Table Association between serum Mg and outcome, results of Cox proportional hazards models^#^. (DOC)

S2 Table Sensitivity analysis of the association between serum Mg and outcome. (DOC)
