# Supplementary material for: Serum Magnesium and Sudden Death in European Hemodialysis Patients
Source: PLoS One. 2015 Nov 23;10(11):e0143104. doi: 10.1371/journal.pone.0143104 (PMC4658157; doi:10.1371/journal.pone.0143104)
Supplement: S1 Table — (DOC) [file pone.0143104.s001.doc]

**S1 Table: Association between serum Mg and outcome, results of Cox proportional hazards models^#^**

|  | All-cause mortality  (137 events) | Cardiovascular mortality  (43 events) | Non-cardiovascular mortality  (94 events) | Sudden death  (24 events) |
| --- | --- | --- | --- | --- |
| Crude* | 0.85  (0.77-0.94)^&^ | 0.73  (0.62-0.85)^&^ | 0.91  (0.81-1.01) | 0.76  (0.62-0.93)^&^ |
| Model 1^ | 0.83  (0.75-0.92)^&^ | 0.72  (0.61-0.84)^&^ | 0.88  (0.78-0.99) | 0.75  (0.60-0.92)^&^ |
| Model 2^$^ | 0.86  (0.77-0.95)^&^ | 0.73  (0.62-0.86)^&^ | 0.92  (0.81-1.04) | 0.74  (0.59-0.93)^&^ |
| Model 3^¶^ | 0.89  (0.80-0.99)^&^ | 0.73  0.62-0.87)^&^ | 0.96  (0.85-1.08) | 0.74  (0.59-0.94)^&^ |
| Model 4^§^ | 0.87  (0.78-0.97)^&^ | 0.73  (0.61-0.87)^&^ | 0.94  (0.83-1.06) | 0.74  (0.57-0.96)^&^ |

Results presented as hazard ratios (HRs [95% confidence intervals]) per 0.1 mmol/L higher baseline serum magnesium concentration. Adjusted for confounders described below with each term entered separately into the model (^#^multivariable Cox regression analysis)

* Crude model (n = 365)

^ Adjusted for age, sex, dialysis vintage, residual kidney function (n = 365)
^$^ Adjusted as in model 1 plus diabetes mellitus, BMI, history of cardiovascular disease and dialysis modality (n = 351)
^¶^Adjusted as in model 2 plus serum albumin, mean pre-dialytic systolic blood pressure and treatment time (n = 339)

^§^Adjusted as in model 3 plus serum calcium, serum parathyroid hormone and serum phosphate (n=334)

^&^ indicates a significant difference in adverse event risk (*p*<0.05)
