# Supplementary material for: Serum Magnesium and Sudden Death in European Hemodialysis Patients
Source: PLoS One. 2015 Nov 23;10(11):e0143104. doi: 10.1371/journal.pone.0143104 (PMC4658157; doi:10.1371/journal.pone.0143104)
Supplement: S2 Table — (DOC) [file pone.0143104.s002.doc]

**S2 Table: Sensitivity analysis of the association between serum Mg and outcome**

|  | All-cause mortality  (116 events) | Cardiovascular mortality  (39 events) | Non-cardiovascular mortality  (72 events) | Sudden death  (21 events) |
| --- | --- | --- | --- | --- |
| Crude* | 0.84 (0.75-0.93)^&^ | 0.70 (0.59-0.83)^&^ | 0.95 (0.84-1.07) | 0.75 (0.61-0.93)^&^ |
| Model 1^ | 0.85 (0.76-0.95)^&^ | 0.71 (0.60-0.84)^&^ | 0.96 (0.85-1.08) | 0.76 (0.62-0.93)^&^ |
| Model 2^$^ | 0.84 (0.76-0.94)^&^ | 0.70 (0.59-0.83)^&^ | 0.94 (0.83-1.06) | 0.76 (0.61-0.95)^&^ |
| Model ^¶^ | 0.88 (0.79-0.98)^&^ | 0.72 (0.60-0.85)^&^ | 0.96 (0.89-1.02) | 0.79 (0.65-0.96)^&^ |
| Model 4^§^ | 0.86 (0.79-0.97)^&^ | 0.71 (0.60-0.85)^&^ | 0.95  (0.89-1.03) | 0.79  (0.66-0.96)^&^ |

Results presented as hazard ratios (HRs [95% confidence intervals]) per 0.1 mmol/L higher baseline serum magnesium concentration. Adjusted for confounders described below with inverse probability weighting, censoring at the time of discontinuation of treatment

* Crude model (n = 365)

^ Adjusted for age, sex, dialysis vintage, residual kidney function (n = 365)
^$^ Adjusted as in model 1 diabetes mellitus, BMI, history of cardiovascular disease and dialysis modality (n = 351)
^¶^Adjusted as in model 2 serum albumin, mean pre-dialytic systolic blood pressure and treatment time (n = 339)

^§^ Adjusted as in model 3 plus serum calcium, serum parathyroid hormone and serum phosphate (n = 334)

^&^ indicates a significant difference in adverse event risk (*p*<0.05)
